# Engaging high schools for the co‐creation of hands‐on teaching resources for medical programmes

**DOI:** 10.1111/medu.14892

**Published:** 2022-08-18

**Authors:** Christian Moro, Charlotte Phelps

## 1 WHAT PROBLEMS WERE ADDRESSED?

Most medical school physiology and anatomy laboratories house a substantial number of hands‐on teaching resources, including silicon‐based models, cadaveric tissues and pathological specimens. However, these are limited by solely depicting a single healthy or diseased state with no ability to show variations. This leaves tertiary educators limited in their capability to offer ‘hands‐on’ examples of important disorder presentations. In recent years, high schools have undergone exponential growth in their employment of technology, and many now host engineering societies, information technology groups and STEM‐based activities. Linking up with local secondary schools presents an ideal opportunity to engage school students in the co‐creation of high‐quality, accurate and hands‐on resources that can be used within medical programme teaching. If structured correctly, this endeavour can be performed in a way that benefits both high school and university students.

## 2 WHAT WAS TRIED?

We assessed limitations between the teaching requirements and available models in our medical programme's laboratories. For example, it would be helpful to show the natural progression of a fatty heart, as well as a brain lesion in multiple sclerosis, neither of which were available from the current resources. This would enable an educator to hold up the model in class, point to important features and then pass it around for group examination, something not possible when using more modern teaching interventions such as virtual reality.^1^ To create these hands‐on resources, we engaged with local schools to 3D print organ models, assess these for accuracy, and then paint them with colourful, acrylic‐based paint. Under supervision by a tertiary academic, staff and students from the: Information Technology department helped create 3D model computer files; Engineering department 3D printed the models; Science department assessed the printed model's accuracy; Arts department chose the correct painting supplies and worked with Biology students to correctly paint the models. Although multiple staff were involved, the individual time commitment from each was only 1–2 h per project. In most cases, two identical models were created concurrently with the school keeping one, and the other handed to the university's medical programme. Students could then utilise this hands‐on teaching resource for learning.

## 3 WHAT LESSONS WERE LEARNED?

In many cases, tertiary institutions assist local schools through guest presentations, awarding prizes, and offering campus visits. Inversely, through this project, school students assisted our medical programme. Many high schools have a wide range of resources, such as 3D printers and computer expertise, that can add great value to the creation of teaching resources. Although in some cases virtual 3D models using a computer or tablet may be able to showcase diseases,^1^ there is still a benefit in offering hands‐on physical specimens for learning within the laboratory environment. When the final products were in use, educators, medical and biomedical sciences students commented that they appreciated and enjoyed having access to life‐sized physical models to hold during in‐class lessons.
